# Dentistry in the Exposition

**Published:** 1874-12

**Authors:** 


### DENTISTRY IN THE EXPOSITION.

To the Editor of the Dental Register:

Taking a natural interest in science because perhaps that it is more or less involved in mystery to the unintiated, I examined every case of scientific apparatus and surgical and dental instruments exhibited in the recent Industrial Exposition of Cincinnati, with the indescribable curiosity peculiar to the mere seeker after knowledge, and was really puzzled to know whether a city so renowned as Cincinnati in the annals of manufacture, and an occasion so national in character

*There were six teeth filled. Ed.

as the Industrial Expositon could develop nothing better in mechanical dentistry than the few sets of artificial teeth kept in chewing motion by clock-work. I wondered if this was all the city or the country could show to convince the world of the superiority and excellence of American dentistry and and concluded that if it really was, there is no longer any ground for blaming people for the horrid appearance of their mouths and pitiful attempts at speech they make after going through the steam dental mills advertised in the news papers and represented at public shows, by the specimens of work alluded to.

I am free to confess that I know nothing about dentistry except from observation. I know a good job of filling especially after I have been assured that it has been chewed on for fifteen or twenty years and is just as good as when first put in, but I am perfectly confident that I can tell a first class job of artificial dentures the moment the wearer opens his mouth to speak, or when he eats. In the first place they fit the mouth and present no undue proportion to the features, that is, they are neither too large nor too small; they suit the complexion, follow the facial lines and, in a word convey an impression to the beholder that they are firm set and belong to the mouth. In very fact they do not convey the impression of artificiality but on the contrary impress the beholder that they are natural.

Now, how intelligent men and women can go into a dentist's establishment and have a set of store teeth put into their mouths and pay for them and come away with the delusion that they are suited is quite past my comprehension. Yet the thing is of frequent occurence. I can imagine the art of the dentist in effecting the sale. It recalls the amusing scene depicted by the negro minstrel of the country man and the slopshop clothes dealer. The ruralist is encased in a coat and stood up before the looking glass. Old clo' gathers the super-abundant cloth of the brest in his hand and directs the attention of Mr. Greeny to the "neat fit" of the back and then turning him round for a view of the breast fit grabs the garment by the back and makes it smooth and nice in front accompanying this sleight-of-hand performence with reiterated assurances that, "that coat fits you mein friendt so bedder as though it never vos madt for you, so help me grachious. I pledge you mien vordt that that coat cost me twenty-nine thallers but I tell you vot I do I sells you that coat for eighteen thallers, unt I wont take one cent less, so help me grachious, how much'll ye give?"

The man that sells store teeth has just about as much true art in fitting and conscience in selling his wares as old clo' and yet it is only the few who see through him and his cheap dentistry even in this country famous the world over for superior work. And it is because America enjoys the singular reputation for superiority that I felt astonished that the great city of Cincinnati and her representative Industrial Exposition had nothing to show the world in the way of dentistry, but half a dozen sets of store teeth mounted on rubber and set in motion by clock work to catch the eye and provoke the wonder of the bumpkins from the country.

Of course I can not account for the deficiency except that superior workmen disdained the field either because it was too small, or that the art of the mechanical dentist really cannot be exhibited in the simple work of mounting a set of teeth on rubber. B.

